# Optimization of Ultrasonic-Assisted Incremental Sheet Forming

**DOI:** 10.3390/ma17133170

**Published:** 2024-06-28

**Authors:** Ngoc-Tuan La, Quoc-Huy Ngo, Van-Dam Vu, Thu-Ha Mai, Ky-Thanh Ho

**Affiliations:** 1 Faculty of Mechanical Engineering, Vinh University of Technology Education (VUTE), Vinh City 430000, Vietnam; langoctuan.ktv@gmail.com; 2 Faculty of Mechanical Engineering, Thai Nguyen University of Technology (TNUT), Thai Nguyen 250000, Vietnam; ngoquochuy24@tnut.edu.vn; 3 Faculty of Engineering and Technology, University of Information and Communication Technology—Thai Nguyen University (ICTU), Thai Nguyen 250000, Vietnam; vudam@tnu.edu.vn; 4 Thai Nguyen High School for Gifted Students, Thai Nguyen 250000, Vietnam; maiha180184@gmail.com

**Keywords:** ultrasonic, incremental, forming, reduction force

## Abstract

Implementing the ultrasonic vibration-assisted incremental sheet-forming (UISF) process has been proven to significantly reduce the forming force, improve the surface quality, and enhance the accuracy of the sheet-forming process. However, such effectiveness has primarily focused on easily deformable materials (such as AA1050 and AA1060 aluminum alloys) and small step-down sizes (from 0.3 mm to 0.5 mm). To further enhance the process, it is crucial to study larger step-down sizes and harder materials. In this study, a series of UISF experiments were conducted, with step-down sizes ranging from 0.5 mm to 1.5 mm and feed rates ranging from 200 mm/min to 1200 mm/min. The influence of ultrasonic vibration on the effectiveness of force reduction and the optimal operation parameters was experimentally tested. Forming aluminum alloy AA5052, a difficult-to-deform material with two thicknesses of 0.5 mm and 1.0 mm, indicates that the axial force *Fz* and the tool movement resistance force *Fy* tend to decrease significantly with ultrasonic vibration assistance. Optimal equations for force reduction *Fz* and *Fy* have been developed for plate thickness based on the step-down size and feed rate. The optimal results show that for 1.0 mm thickness, reductions in *Fz* and *Fy* can reach 58.73% and 69.17%, respectively, and that of 64.17% and 71.98%, respectively, for 0.5 mm thickness.

## 1. Introduction

The incremental sheet-forming (ISF) process, first announced in 1967 by Leszak Edward [1], is an ideal method for forming sheet products without a mold, applying to shape profiles from simple to complex. Using the ISF process, product sizes can change flexibly, from small to large. During the forming process, only a part of the product is in direct contact with the forming tool, so the main deformation force components are small [2]. This process is superior to the conventional sheet stamping and drawing processes used in small production scenarios [3,4]. In addition, compared to conventional sheet stamping and deep drawing processes, the ISF process does not require the fabrication of complex, expensive molds [3,5,6,7]. Additionally, it provides more flexibility in choosing machine tools to shape products [4,8,9] and more flexibility when changing products [7]. Due to these outstanding advantages, the ISF process is considered to have great potential for technologies in many fields, such as manufacturing automobile body panels, household appliances, rapid prototyping, and manufacturing replacement tissue applications in the field of medicine [4,5,10,11,12,13,14].

To improve the applicability of ISF technology, a number of studies have been performed. Some scientists have conducted research on residual stress and its effects on product quality after forming [15,16]. Some new models were proposed to predict forming forces [17,18]. The influence of contact friction on the temperature and deformability of s during forming via the ISF process was researched and evaluated [19]. The characteristics of crystal grain deformation during the ISF process were determined [20]. The optimal parameters for forming AA5052 aluminum alloy sheet product were determined [21,22]. The springback behavior of metal plates and metal/polymer laminates after forming with the ISF process was researched, and models were proposed for prediction [23,24]. The effects of process parameters on energy consumption for the ISF process were studied [25,26]. A surface roughness prediction model for incremental sheet metal-forming parts was established [27]. Flexible molds to improve the quality of shaped products using the ISF process were used [28]. The deformation ability in multiple steps with different tool path trajectories was studied [29].

Studies on deformation force behavior play an important role in explaining the plastic deformation mechanism, predicting failure, and controlling and optimizing the deformation process. Increasing the values of input parameters, such as step-down size, tool diameter, wall inclination angle, and sheet thickness, along with the application of high-strength sheet metals and lightweight alloys, leads to increasing the forces acting on the forming tool. In addition, improving the ISF efficiency by decreasing the springback and surface roughness of the product is an important challenge in industrializing the process. Consequently, many technologies have been exploited to reduce deformation force to improve the ability to form sheet products processed by the ISF method, such as deformation using hot state [30,31,32], hot electric state [33], magnetic field-assisted [34], and ultrasonic-assisted methods [35,36]. Among these, the exploitation of ultrasonic-assisted ISF (called UISF) or ultrasonic vibration to assist ISF (called UV-ISF) has attracted much interest in sheet metal-forming research. The phenomenon of reducing yield stress and softening materials by ultrasonic vibration was first discovered by Langnecker in 1955 [37]. Since then, ultrasonic vibration has been researched to assist in many different manufacturing processes, such as milling [38,39], drilling [40,41,42], deep drawing [43], forging [44], and extrusion [45,46].

The UISF process has garnered significant attention due to its potential benefits. Research on UISF has demonstrated the effectiveness of ultrasonic vibration in reducing deformation force (up to 60% of the forming force in the horizontal direction, and up to 50% of the forming force in the vertical direction) [47,48,49], improving surface quality [50,51], and enhancing the accuracy of products [47,49,50,52]. However, it is important to note that most existing studies have primarily concentrated on soft materials with exceptional plastic deformability, such as aluminum alloy AA1050-O [35,47,51,52,53,54,55,56,57] or AA1060 [58]. Moreover, previous experiments were performed with small step-down sizes, usually from 0.3 mm to 0.5 mm, which are much smaller than those of typical ISF processes [4,7]; thus, the production time would not be reduced significantly. Meanwhile, some studies have implemented multi-stage forming to reduce the plastic deformation force while increasing accuracy and improving the surface quality [47,56]. The effects of ultrasonic vibration on the force reduction have not yet been fully evaluated because of the limited number of experiments. Furthermore, there have not been many studies applying ultrasonic assistance to the ISF process for harder and more difficult-to-shape materials, such as AA5052-H34 [59], AA5052 [50], AA6061-T6 [60] aluminum alloy, or Q235 steel [61]. Similar to other studies, these studies also applied small step-down sizes in experiments. Due to the limited number of experiments [51], optimizations to determine the best parameters of ISF using ultrasonic assistance have also rarely been found.

In this work, the optimal selection of input parameters to reduce the forming force using the ultrasonic-assisted ISF process is a major objective. In addition, to reduce forming time, in this study, larger step-down sizes than those in the recently published studies on the ultrasonic vibration-assisted ISF process [35,47,51,52,53,54,55,56,57,58] were selected for investigation. In addition to proposing new machining parameters, the implementation of the ultrasonic-assisted vibration ISF process is applied to AA5052 aluminum alloy, a specific type of material popular for sheet metal forming.

Experiments were designed and scientifically controlled to optimize the process and help manufacturers choose the appropriate operation parameters. The ultimate objective is to improve the applicability of the UISF process in practical applications.

## 2. Materials and Methods

### 2.1. Experimental Setup

**Figure 1:**
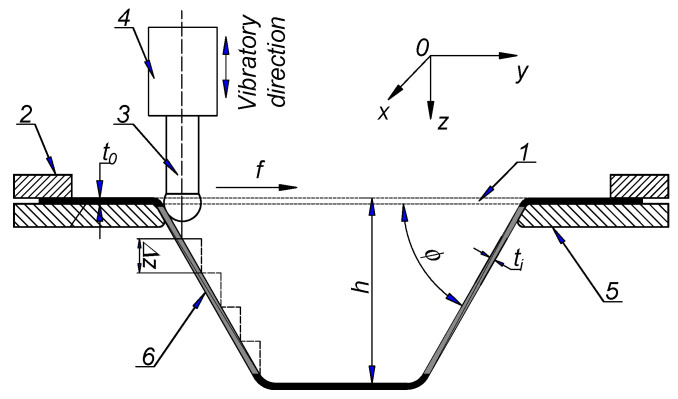
Schematic of the UISF process.

Figure 1 illustrates a schematic of the ultrasonic-assisted incremental sheet-forming (UISF) process. The initial workpiece (1) with thickness *t*_0_ is clamped between blank holder (2) and back plate (5). Strategy Geometry/shape of the forming tool (3) was selected in accordance with the product profile (e.g., forming depth *h* and wall angle *ϕ*) to be shaped by using a CNC machine or robotic arms with the tool path programmed based on the part profile and generated by the CAD/CAM program [4]. To form the full depth *h*, the tool advances axially in steps with step-down size Δ*z* and moves in horizontal direction with feed rate *f*. The tool usually has a hemispherical-head tool with a diameter commonly ranging from 5 mm to 20 mm [7,62]. In UISF process, the tool is connected to an ultrasonic transducer (4), which makes it vibrate in the vertical direction. At the end of the processing process, the final product (6) with thickness *t_i_* is received. It should be noted that the tool may or may not rotate. Previous studies show that the rotation speed of the tool has almost no effect on the forming force components [49,52,63]. In this study, the forming tool was selected not to rotate in all experiments.

**Figure 2:**
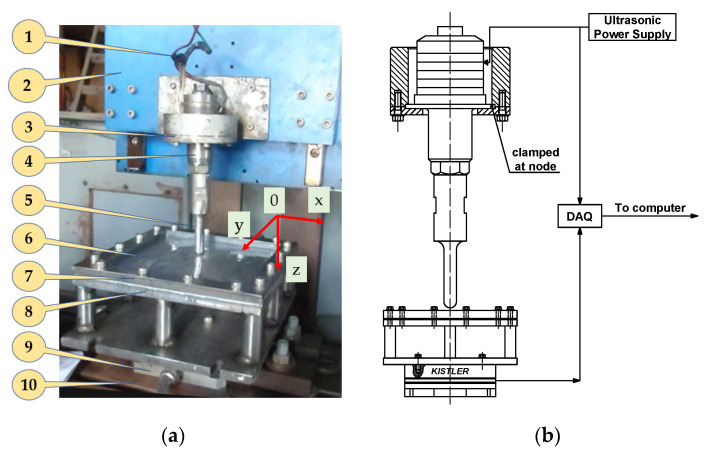
Practical experimental setup: (**a**) a front view and (**b**) transducer clamping diagram and model for collecting experimental parameters: (1) ultrasonic supply; (2) head of CNC machine; (3) clamp; (4) ultrasonic transducer; (5) forming tool; (6) workpiece plate; (7) blank holder; (8) back plate; (9) Kistler 3-component dynamometer; (10) table bed of machine.

The experimental arrangement is shown in Figure 2. In this study, a CNC machine, converted from a VHR AP vertical NC milling machine from Shizuoka (Japan), was used. In particular, the drive motors of 03-axis were replaced by three 3-phase AC servo hybrid electric motors, each motor having a maximum torque of 12 Nm. The forming programs can be input directly on the machine or imported via USB port. To determine the force components during deformation, a Kistler 3-component dynamometer type 9257B (Kistler, Winterthur, Switzerland) was used. The ultrasonic vibrations were implemented by means of an ultrasonic generator MPI WG-3000 WG (M P Interconsulting, Le Locle, Switzerland) and a transducer Herrmann KHS20-IP50-L (Herrmann Ultraschalltechnik GmbH & Co. KG, Karlsbad, Germany), working at 20 kHz frequency. The deformation tool has a diameter *d* of 14 mm, made from high-speed steel. It should be noted that the length and structure of the deformable tool are designed to ensure that the vibration amplitude at the tip of the tool is maximum. After carefully calculating the wave propagation theory to obtain a deformation tool size suitable for the frequency of the ultrasonic transducer and the generator [35,36], the ultrasonic impedance V-I method was used to scan the system’s resonant frequency [64]. To ensure the system works properly and does not lose vibrations transmitted from the transducer to the tool, the transducer must be clamped tightly at the “node” (as shown in Figure 2). According to the principle of ultrasonic wave propagation, “node” is the position at which the vibration amplitude of the transducer or tool is zero [35,36,64]. The results show that the working frequency of the ultrasonic working system is 20.002 kHz, equal to the resonance frequency, as announced by the ultrasonic transducer manufacturer. During the forming process, ultrasonic vibration was supplied to the working system in turning on/off mode, in which turning-on means the system works with assisted ultrasonic vibration, and, on the contrary, turning-off means the system works without the assistance of ultrasonic vibration. The NI SignalExpress software (version 2015) was employed to collect data of forming forces and current supplied to the transducer. In this study, a DAQ NI-6210 (National Instruments, Austin, TX, USA) was used as a data collector, such as forming force signals and current signal supplied to the transducer. The sampling frequency was 1000 times/s for all experiments.

Different from the previous experiments using soft materials, this study proposes using AA5052 aluminum alloy, a harder material for the metal-forming process. With greater yield strength and a higher ability to harden by cold deformation (compared to AA1050 and AA1060 aluminum alloys, as shown in Table 1), AA5052 aluminum alloy is commonly used for inner body and trim panels in the automotive industry, vehicle components, ships, and vessels [65]. Due to these characteristics, plastically forming AA5052 is more challenging than forming AA1050 and AA1060 aluminum alloys [47,59,65,66,67,68].

In this study, two different types of plate, with thicknesses of 0.5 mm and 1.0 mm, were used. To clamp the sheets into the mold, these plates were cut into square shapes with dimensions of 240 mm × 240 mm.

### 2.2. Design of Experiments

**Figure 3:**
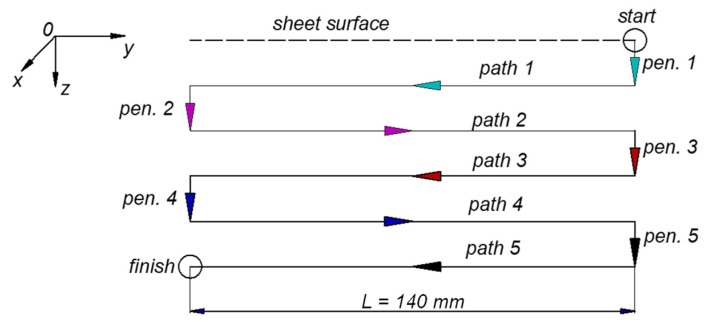
The forming tool path strategy (in which pen. is penetration).

Studies on the ISF and UISF processes show that, in the early stages of the forming process, the main force components (in vertical and horizontal directions) increase rapidly. However, when the deformation depth reaches a certain limit, these forming force components will reach a definite and stable limit. Beyond this point, the forces remain unchanged, even when the depth is increased [47,55,69]. This behavior is explained by the balance between wall thinning (which reduces force) and strain hardening (which increases force). In addition, the tool movement trajectory also affects the shaping force and quality of the product [14,70]. There are many different types of tool movement trajectories, such as spatial or contour [71]. In this study, the forming tool path strategy is chosen as shown in Figure 3 below.

As shown in Figure 3, at the beginning of the forming process, the tool penetrates the sheet surface by a step-down Δ*z* (in the vertical direction *Oz*), then moves horizontally *Oy* at a certain feed rate *f* (see the coordinate system in Figure 1). This process is repeated five times to create a straight groove. During the experiment, ultrasonic vibrations are supplied to the tool through the transducer in on/off mode to evaluate the influence of applying ultrasonic assistance. The deformation force components *Fz* (in vertical direction), *Fy* (in the horizontal direction of the forming tool), and *Fx* and the electrical signal supplied to the transducer are recorded for processing.

Previous studies on ISF [4,7,63] show that there are many technological parameters that affect deformation forces, forming quality, and surface quality, such as deformation tool diameter *d*, feed rate *f*, tool rotation speed *n*, forming trajectory, axial step-down size Δ*z*, forming depth *h*, wall angle *ϕ*, plate thickness *t*. In particular, feed rate *f* and step-down size Δ*z* are two parameters that have a strong influence on the deformation force components as well as the shaping ability and product surface quality. When adding ultrasonic assist to the ISF process, the deformation forces, forming quality, and surface quality are also affected by ultrasonic parameters. This study focused on evaluating the effect of major parameters, including tool feed rate *f* and step-down size Δ*z*, on the amount of deformation force reduction for *Fz* and *Fy* in two stages, with and without the ultrasonic-assisted ISF process. Other parameters were kept constant during the experiments. All experiments were performed under lubricating oil conditions to minimize contact friction force between the plate surface and the tool surface.

In this study, two experimental designs were implemented: comparative experiments and optimization experiments. The comparative experiments were built to conduct a paired T-test to evaluate the reduction in *Fz* and *Fy* forces with ultrasonic assistance in ISF, as compared to conventional ISF without ultrasonic assistance. This was carried out to determine the effectiveness of the ultrasonic assistance in reducing the *Fz* and *Fy* forces during the forming process. The optimization design was implemented to carry out optimal forming parameters to obtain the largest forming force reduction. Minitab 18^®^ software was used to design experimental plans as well as to analyze the collected data.

In the comparative study, a hypothesis test for the mean difference between paired observations of the two ISF stages (with and without ultrasonic assistance) was applied. Experimental parameters of the step-down size Δ*z*, the feed rate *f*, and the sheet thickness *t* were varied but kept consistent within each pair between the two stages. The paired T-test was then applied for analyzing differences between pairs, i.e., differences with and without ultrasonic assistance on the same ISF parameters. The following statistic hypothesis was proposed:

(1) $$\left\{\begin{array}{l} H_{0}:\mu_{0}=0\\ H_{1}:\mu_{0}<0 \end{array}\right.$$

where *µ*_0_ is the mean difference between the forces induced in UISF and ISF, calculated with a 95% confidence interval. Once the alternative H_1_ is accepted (*µ*_0_ < 0), the *Fz* and *Fy* from UISF can be considered statistically smaller than that from ISF.

In order to implement the paired T-tests, a two-level experimental design was built with parameter variables shown in Table 2.

The response surface methodology using face-centered CCD was applied to find the optimal settings for the selected parameters. Face-centered designs are a type of central composite design where the axial points or “star” points are at the center of each face of the factorial space, so the axial levels = ±1. This variety of design requires 3 levels for each factor, as depicted in Table 3. The two investigated parameters were selected based on the results of preliminary experiments (see Section 3.3 below). The optimal process was applied separately for sheet thicknesses of 0.5 mm and 1.0 mm.

The results obtained and detailed analysis are represented in the next section.

## 3. Results and Discussion

### 3.1. Preliminary Tests

**Figure 4:**
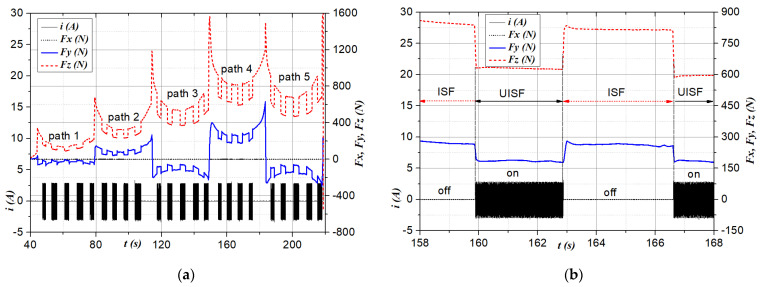
Deformation force *Fx* (N), *Fy* (N) and *Fz* (N) at conditions: *t* = 1.0 mm, Δ*z* = 1.5 mm, *f* = 200 mm/min: (**a**) total 5 paths; and (**b**) from 158 s to 168 s in the path 4.

**Figure 5:**
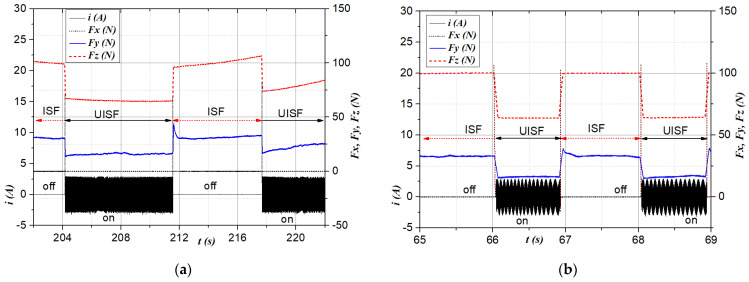
Deformation force *Fx* (N), *Fy* (N) and *Fz* (N) at conditions: (**a**) path 4 from 202 s to 222 s, *t* = 0.5 mm, Δ*z* = 1.0 mm, *f* = 200 mm/min; (**b**) path 5 from 65 s to 69 s, *t* = 0.5 mm, Δ*z* = 0.5 mm, *f* = 700 mm/min.

The influence of ISF-assisted ultrasonic vibration was initially evaluated by observing the change in deformation force upon switching the ultrasonic transducer’s power supply on and off. Figure 4 and Figure 5 represent two typical cases of the force *Fz* (in red short dash line), *Fy* (in blue solid line), and *Fx* (in black short dot line) with respect to the step-down size Δ*z* and feed rate *f,* corresponding to two thicknesses *t* of 0.5 mm and 1.0 mm, respectively. In those figures, the current supplied (*i*) to the transducer is recorded and shown as a solid black line for reference.

Figure 4a depicts the variation in the deformation force component *Fx, Fy*, and *Fz* for four strokes. A zoomed-in view for one stroke is shown in Figure 4b. It can be seen in Figure 4a that, for each moving tool stroke, the force components tend to be smallest in the middle (as similar to valleys) and largest at the end and beginning of the stroke (formed as peaks). This is because, the closer to the clamping part, the less plastic deformation ability, requiring force to cause greater plastic deformation [55]. The middle part of the stroke represents a stable deformation, more consistent with actual forming conditions, especially in the case of the tool that moves along spiral paths or large parts. Figure 4 and Figure 5 show that the force component *Fz* (in the vertical direction) is much larger than the force component in the horizontal direction *Fy* (the direction that hinders the forward motion of the forming tool). Meanwhile, the results show that *Fx* does not change during the forming process. Compared with the previous path, after the forming tool further penetrates the workpiece surface and step Δ*z*, both force components *Fz* and *Fy* tend to increase (as shown in Figure 4). This can be explained by the cause of hardening during deformation in the cold state [36,53]. Two different experimental conditions with step-down sizes 1.0 mm and 0.5 mm are shown in Figure 5a and Figure 5b, respectively. From Figure 4 and Figure 5, it is evident that the application of ultrasonic vibration (annotated by “on” for the current signal *i*) results in a significant decrease in the deformation force *Fz* and *Fy* compared to the results obtained without ultrasonic assistance (annotated by “off”), for both investigated sheet thicknesses. This can be observed across all strokes, indicating the effectiveness of ultrasonic vibration in reducing deformation force *Fz* and *Fy*. The reduction in force *Fz* is explained by the fact that ultrasonic vibration has the effect of softening the material, that is, reducing the deformation resistance, making the material flow more easily [62]. For *Fy*, the force reduction could be due to two reasons: (1) the material softening due to ultrasonic vibrations [36,53,62] and (2) ultrasonic vibration improving the lubrication conditions [72,73], thereby reducing friction between the tool and the workpiece surface.

**Figure 6:**
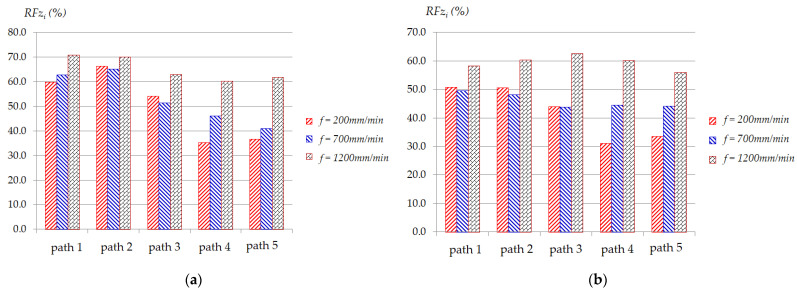
Effects of input parameters on average of reduction force *RFz_i_* (%) in five paths at conditions: (**a**) *t* = 0.5 mm, Δ*z* = 0.5 mm; (**b**) *t* = 1.0 mm, Δ*z* = 0.5 mm.

**Figure 7:**
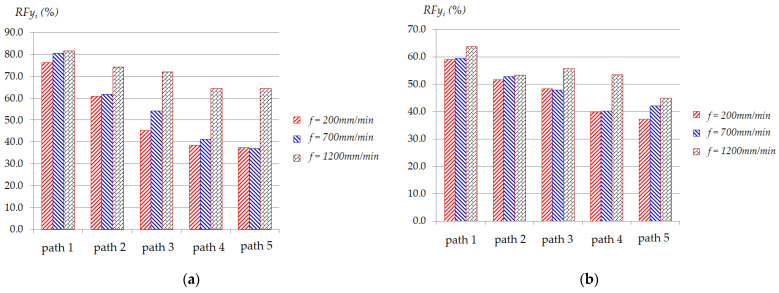
Effects of input parameters on average of reduction force *RFy_i_* (%) in five paths at conditions: (**a**) *t* = 0.5 mm, Δ*z* = 1.5 mm; (**b**) *t* = 1.0 mm, Δ*z* = 1.5 mm.

Figure 6 and Figure 7 present typical statistical results of *RFz_i_* (%) and *RFy_i_* (%) in five paths under some specified conditions, respectively, in which *RFz_i_* (%) and *RFy_i_* (%) are calculated according to Equation (2) and Equation (3), respectively (see Section 3.3 below). These two figures show that the greater the thickness, the lower the effectiveness of *RFz_i_* (%) and *RFy_i_* (%). Figure 6 shows that with different feed rates, *RFz_i_* (%) is significantly different. The larger the feed rate, the greater the *Fz* force reduction effect. As depicted in Figure 6a, for the smaller thickness, after each path, *RFz_i_* (%) tends to decrease. At low feed rates (i.e., *f* = 200 mm/min and *f* = 700 mm/min), the decreasing trend of *RFz_i_* (%) is larger than that at high feed rate (*f* = 1200 mm/min). However, for the thickness of 1.0 mm, as shown in Figure 6b, the decreasing trend of *RFz_i_* (%) after each path at feed rates of 700 mm/min and 1200 mm/min is not clear. This can be explained because when the feed rate is raised, the heat from contact friction between the surface of the plate and the tool increases, making the material softer [62]. The *Fy* force reduction effect during five paths, as depicted in Figure 7, also shows the same trend as *Fz* force reduction.

### 3.2. Paired T-Tests

**Figure 8:**
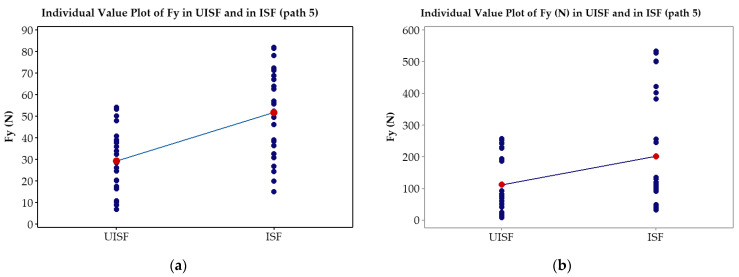
Paired T-test plots *Fy* in UISF and in ISF processes: (**a**) 0.5 mm in thickness and (**b**) 1.0 mm in thickness.

**Figure 9:**
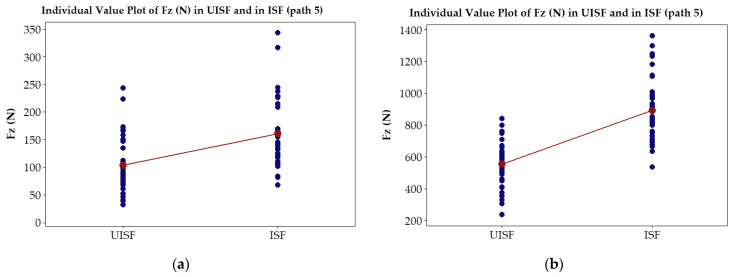
Paired T-test plots *Fz* in UISF and in ISF processes: (**a**) 0.5 mm in thickness and (**b**) 1.0 mm in thickness.

To assess the effectiveness of ultrasonic assistance on the reduction in *Fz* and *Fy* forces, 78 tests for the paired T-test analysis were implemented, with 39 tests for the stage of ultrasonic-assisted ISF and the remaining tests for ISF stage without ultrasonic assistance. In each stage, experimental parameters described in Table 2 were applied. Figure 8a compares the force distribution *Fy* average in path 5 for the 0.5 mm thickness, while Figure 8b shows the result in path 5 for the 1.0 mm thickness. Similarly, Figure 9a compares the average force distribution *Fz* in path 5 for the 0.5 mm thickness, and Figure 9b shows the result in path 5 for the 1.0 mm thickness. Our findings indicate that at the same path, the average force reduction *Fy* for the 0.5 mm thick plate is 43.6%, while for the 1.0 mm thick plate, it is 44.8%. Meanwhile, at the same path, the average force reduction *Fz* for the 0.5 mm thick plate is 35.6% and 38.0% for the 1.0 mm thick plate. It should be noted that this reduction is only calculated in path 5, which has the largest deformation force component *Fz* and *Fy* during the investigated experiment. Additionally, the study results in path 5 reveal that the distribution range forces in the UISF process are narrower than that in the ISF process.

With all *p*-values, which are much smaller than 0.05 (as depicted in Table 4 and Table 5), it can be confirmed that the force induced from UISF is significantly smaller than those from ISF. Also, the statistical interference with 95% confidence intervals, which do not include any zero values, shows that there are significant differences between the two populations’ means. In other words, the forces induced from UISF are smaller than those from ISF.

### 3.3. Effects of Forming Parameters

From the above investigated results, it can be seen that both *Fz* and *Fy* decreased strongly when applying ultrasonic vibration assistance. Also, it is clear that the axial force component *Fz* is much larger than the component that resists tool motion *Fy*. Therefore, evaluating the effectiveness of this force component will bring more practical meaning. With this research model (see Figure 3), the force component *Fy* affects the energy consumption during the forming process. In order to evaluate the effectiveness of applying ultrasonic assistance, reduction forces for each path were introduced, calculated as a ratio of forces in percentage, as follows:

(2) $$R{Fz}_{i}\;\left(\%\right)=\frac{{Fz}_{ISF}-{Fz}_{UISF}}{{Fz}_{ISF}}*100\%\;$$

(3) $$R{Fy}_{i}\;\left(\%\right)=\frac{{Fy}_{ISF}-{Fy}_{UISF}}{{Fy}_{ISF}}*100\%\;$$

where *Fz_ISF_* and *Fy_ISF_* are the average forming forces when turning off ultrasonic vibration, and *Fz_UISF_* and *Fy_ISF_* are the average forming forces when turning on ultrasonic vibration, respectively. In this study, the values of force components *Fz_ISF_*, *Fz_UISF_*, *Fy_ISF_*, and *Fy_UISF_* are calculated as the average values in the middle of each path, when the ultrasonic vibration is turned off and turned on, respectively.

The average reduction in forming forces *Fz* and *Fy* over five paths (denoted as *RFz* (%) and *RFy* (%), respectively) is calculated as:

(4) $$RFz\;\left(\%\right)=\frac{{RFz}_{1}\;\left(\%\right)+{RFz}_{2}\;\left(\%\right)+{RFz}_{3}\;\left(\%\right)+R{Fz}_{4}\;\left(\%\right)+{RFz}_{5}\;\left(\%\right)}{5}\;$$

(5) $$RFy\;\left(\%\right)=\frac{{RFy}_{1}\;\left(\%\right)+{RFy}_{2}\;\left(\%\right)+{RFy}_{3}\;\left(\%\right)+R{Fy}_{4}\;\left(\%\right)+{RFy}_{5}\;\left(\%\right)}{5}\;$$

The analyzed results are presented in Table 6.

**Figure 10:**
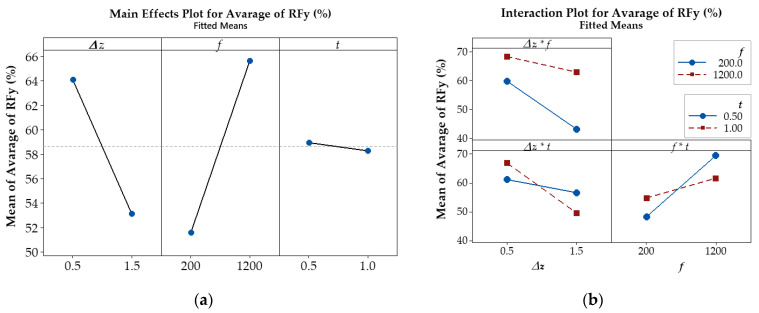
(**a**) Main effect plot; (**b**) interaction effect of parameters on the *RFy* (%).

**Figure 11:**
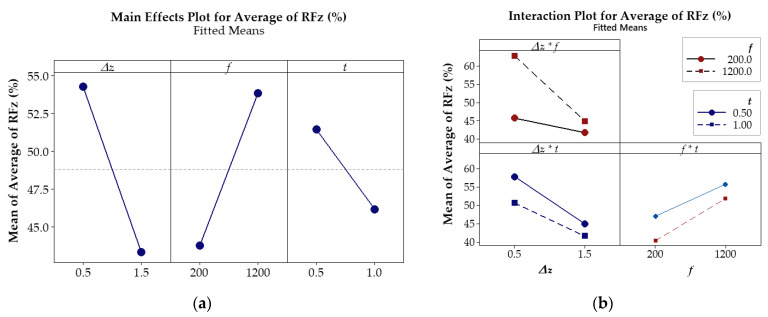
(**a**) Main effect plot; (**b**) interaction effect of parameters on the *RFz* (%).

The main effects and interaction effects between the investigated parameters are depicted in Figure 10 and Figure 11.

The results shown in Figure 10a indicate that the feed rate parameter, *f*, has the highest effect on the average of *RFy*, as shown by the steep slope increases. As the feed rate *f* increases, the average of *RFy* also increases, which implies that the effectiveness of ultrasonic vibration is reduced as the *RFy* tends to increase; i.e., larger feed rates result in greater effectiveness of ultrasonic vibration in reducing the *RFy*. On the other hand, both the step-down size Δ*z* and the thickness *t* tended to decrease *RFy* as the parameter value increased. The impact of step-down size Δ*z* on the *RFy* is slightly less significant than that of feed rate *f*, but the degree of influence of the sheet thickness parameter, *t*, is relatively low, as indicated by the shallow slope.

The results shown in Figure 11a indicate that the step-down size parameter, Δ*z*, has the highest effect on the average of *RFz*, as shown by the steep slope. As Δ*z* increases, *RFz* decreases, which also implies that the effectiveness of ultrasonic vibration reduces with larger step-down sizes. The same trend is observed with the sheet thickness parameter, *t*, but the degree of influence is relatively low compared to Δ*z*. On the other hand, as the feed rate *f* increases, *RFz* tends to increase; i.e., larger feed rates result in greater effectiveness of ultrasonic vibration in reducing the *Fz* force. However, the impact of feed rate *f* on the *RFz* is slightly less significant than that of Δ*z*.

In Figure 10b and Figure 11b, the interaction plot of the input parameters reveals lines that intersect or tend to intersect (Δ*z* * *f*, Δ*z* * *t*, and *f* * *t*), which indicates that there are interaction effects between Δ*z* and *f*, Δ*z* and *t*, as well as *f* and *t*, on the *RFz* and *RFy*. These effects suggest that the force reduction in *Fz* and *Fy* depends on the interaction between Δ*z* and *f*, Δ*z* and *t*, as well as that between *f* and *t*. For the *RFy*, the interaction effect between Δ*z* and *f* is less than that between Δ*z* and *t* and between *f* and *t*. However, the interaction effect between Δ*z* and *f* on the *RFz* is stronger than that of the remaining pairs of input parameters.

Based on these findings, this study focuses on using the two parameters Δ*z* and *f*, which have the largest effects on the reduction in *Fz* and *Fy* force for the optimal tests. The optimization process was performed for different sheet thicknesses, presented in Section 3.4 below.

### 3.4. Optimal Parameters

Optimization experiments to select appropriate parameters for the UISF forming process were carried out, including four cube point experiments, five center points in cube experiments, and four axial point experiments. The averages of *RFz* (%) and *RFy* (%) results for UISF and ISF processes over five paths are shown in Table 7 for plate thicknesses of 0.5 mm and 1.0 mm.

As mentioned in the preliminary test results, when the tool penetrates an additional step-down size Δ*z* to execute a new path, both *Fz* and *Fy* increase, with the *Fz* increases significantly larger than *Fy*. Increments in *Fz* and *Fy* may cause equipment damage or plate tearing. In addition, a large deformation force means high energy consumption, leading to low energy efficiency in the forming process [25,26]. Hence, optimal parameters play an important role to maximize the reduction in deformation forces.

**Figure 12:**
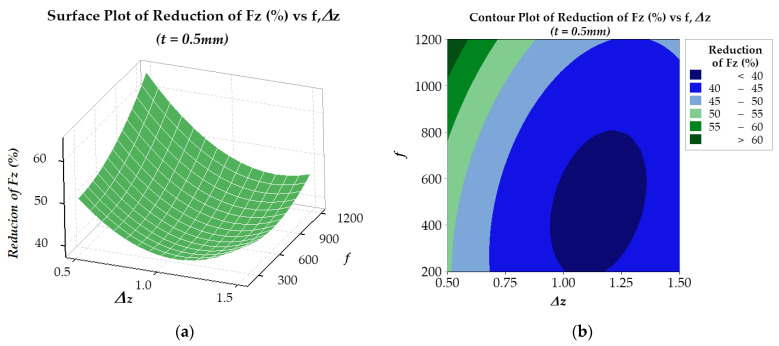
Surface (**a**) and contour (**b**) plots of *RFz* (%) of 0.5 mm thickness plate.

**Figure 13:**
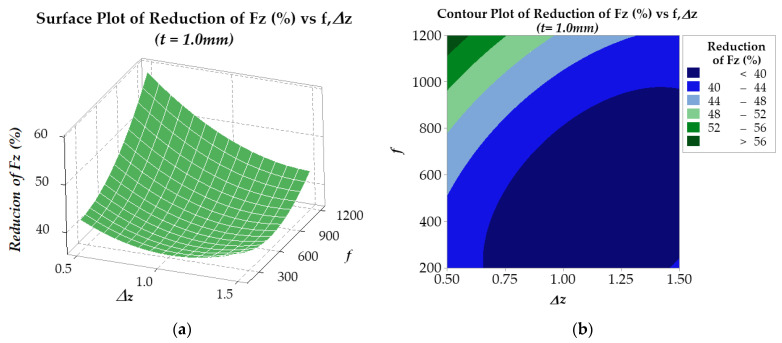
Surface (**a**) and contour (**b**) plots of *RFz* (%) of 1.0 mm thickness plate.

**Figure 14:**
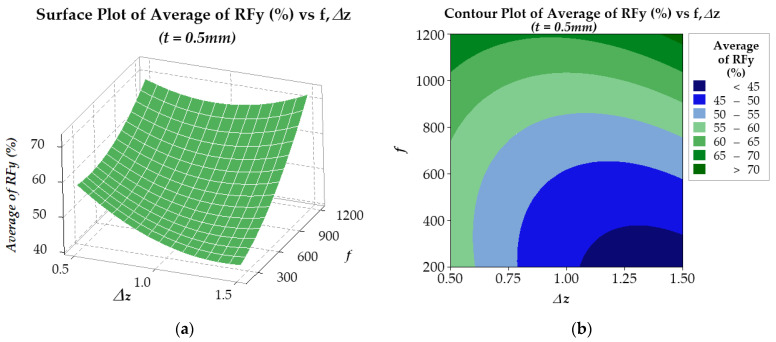
Surface (**a**) and contour (**b**) plots of *RFy* (%) of 0.5 mm thickness plate.

**Figure 15:**
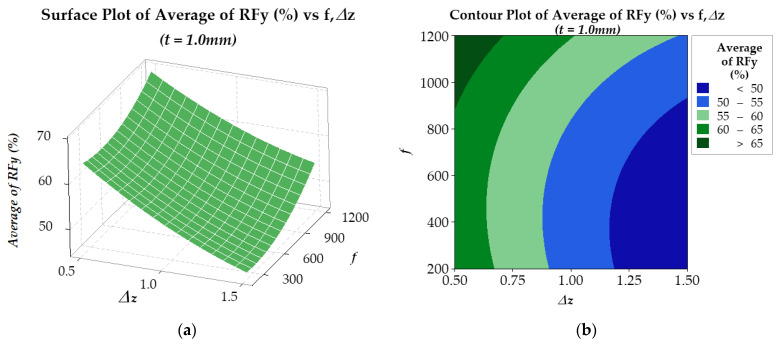
Surface (**a**) and contour (**b**) plots of *RFy* (%) of 1.0 mm thickness plate.

In the optimization process, the response surface method was employed to analyze the *RFz* (%) and *RFy* (%) results. For *RFz* (%), the results are presented in Figure 12 and Figure 13 for two plate thicknesses, 0.5 mm and 1.0 mm, respectively. The findings suggest that for a plate thickness of 0.5 mm (see Figure 12), the regression model R-sq is 98.73% (R-sq(adj) is 97.82%). On the other hand, for a plate thickness of 1.0 mm (see Figure 13), the regression model R-sq is 99.23% (R-sq(adj) is 98.68%). Similarly, the *RFy* (%) results are presented in Figure 14 and Figure 15. With a plate thickness of 0.5 mm (see Figure 14), the regression model R-sq is 91.77% (R-sq(adj) is 85.88%). And for 1.0 mm plate thickness, the regression model (see Figure 15) R-sq is 99.12% (R-sq(adj) is 98.49%). These results demonstrate that the response surface model is an excellent fit for the experimental data and is highly reliable.

The results in Figure 12 and Figure 13 show that the *RFz* (%) is larger when the step-down size Δ*z* is small and the feed rate *f* is high. This phenomenon is because, under the effect of ultrasonic vibration, the temperature at the contact area between the tool and workpiece increases, softening the material and facilitating easier flow [47,48,62]. The higher the feed rate, the greater the heat generated due to contact friction, enhancing the effectiveness of ultrasonic vibration in reducing *Fz* force. With both the sheet thicknesses of 0.5 mm and 1.0 mm, at a step-down size of 0.5 mm, a force reduction *RFz* (%) of about 60% can be obtained with feed rates *f* ranging from 1150 mm/min to 1200 mm/min.

Similar to *RFz*, the results in Figure 15 show that the *RFy* (%) is larger when the step-down size Δ*z* is small and the feed rate *f* is high. However, for smaller thickness *t*, results from Figure 14 show that the effect of Δ*z* on *RFy* (%) is small, and *RFy* (%) depends almost only on the feed rate *f*. That is, for the sheet thicknesses of 0.5 mm, the larger the feed rate, the larger the *RFy* (%).

The equations (in uncoded units) for regressing the *RFz* (%) against the input parameters for the 0.5 mm thick sheet and for the 1.0 mm thick sheet, as shown in Figure 12a and Figure 13a, are presented in Equation (6) and Equation (7) below, respectively:

*RFz_opt_* (%) = 75.95 − 65.13Δ*z* + 0.00119*f* + 30.91Δ*z* × Δ*z* + 0.000013 *f* × *f* − 0.01223Δ*z* × *f* (6)

*RFz_opt_* (%) = 52.95 − 28.13Δ*z* + 0.00183*f* + 14.61Δ*z* × Δ*z* + 0.000015 *f* × *f* − 0.01368Δ*z* × *f* (7)

Similarly, the regression equations of *RFy* (%) for two plates with 0.5 mm and 1.0 mm thickness, from Figure 14a and Figure 15a, are illustrated in Equation (8) and Equation (9) below, respectively:

*RFy_opt_* (%) = 84.91 − 58.8Δ*z* − 0.0222*f* + 19.78Δ*z* × Δ*z* + 0.000017*f* × *f* + 0.01850Δ*z* × *f* (8)

*RFy_opt_* (%) = 80.68 − 32.89Δ*z* − 0.01336*f* + 6.80Δ*z* × Δ*z* + 0.000012*f* × *f* + 0.00399Δ*z* × *f* (9)

The results of solving the optimization problem are shown in Table 8 for *RFz* (%) and Table 9 for *RFy* (%), respectively. Since the objective of this work is to maximize the reduction in forming forces, the target values were selected to be larger than the highest values of *RFz* (%) and *RFy* (%) obtained from the experimental results. The target values for each investigated case are depicted in Table 8 and Table 9.

For the 1.0 mm thickness plate, to achieve the greatest *FRz* (%) and *RFy* (%), the solutions of these two equations are a step-down size of 0.5 mm and feed rate of 1200 mm/min, respectively. With these solutions, the *RFz_opt_* (%) and *RFy_opt_* (%) obtained are 58.73% and 69.17%, respectively. However, for the 0.5 mm thickness plate, to receive an *RFy_opt_* of about 71.98 (%), the results of solving the optimization problem are a step-down size of 1.5 mm and feed rate of 1200 mm/min; i.e., both Δ*z* and *f* are set to their maximum values. With this smaller thickness, to achieve an *FRz_opt_* (%) of 64.17%, the optimal solutions are a step-down size of 0.5 mm and feed rate of 1200 mm/min. This result is significantly improved compared to previous publications when implementing ultrasonic vibration to assist the ISF process [36,53].

## 4. Conclusions

The following remarks summarize our investigation on the effects of ultrasonic vibration and two key process parameters on the reduction in forming force during the ISF process. This study involved forming AA5052 aluminum alloy with two different thicknesses of 0.5 mm and 1.0 mm. A series of ISF processes with and without ultrasonic-assisted experiments, with larger step-down sizes ranging from 0.5 mm to 1.5 mm and feed rates ranging from 200 mm/min to 1200 mm/min, was conducted. Several remarks can be concluded as follows:

- Ultrasonic-assisted ISF experiments were conducted on AA5052 aluminum alloy, a more difficult-to-deform material than used in previous studies.
- During the forming process, the main induced force components, including *Fz* and *Fy*, tend to decrease strongly when applying ultrasonic vibration. These findings demonstrate the positive effect of ultrasonic vibrations on the forming process in general and the ISF process in particular.
- The optimal equations’ operational parameters, including step-down sizes and feed rates, were built for plate thicknesses of 0.5 mm and 1.0 mm.
- For the *RFz* (%), solutions for the optimal problem of two different thicknesses are a step-down size of 0.5 mm and a feed rate of 1200 mm/min. The obtained results show that the optimal value of *RFz_opt_* (%) can reach 64.17% and 58.73% for plate thicknesses of 0.5 mm and 1.0 mm, respectively.
- For the *RFy* (%), the optimal value of *RFy_opt_* for the 0.5 mm thickness plate is about 71.98 (%), with a step-down size of 1.5 mm and a feed rate of 1200 mm/min. For the plate thickness of 1.0 mm, *RFy_opt_* can reach 69.17 (%), with a step-down size of 0.5 mm and a feed rate of 1200 mm/min.

These findings may contribute to expanding the results obtained in ultrasonic-assisted ISF for more difficult-to-deform materials, as well as higher production rates. The results could also be valuable for manufacturers using the ultrasonic-assisted ISF process to select suitable operational parameters to reduce production times while minimizing forming forces.

Further studies should focus on evaluating the surface quality, springback behavior, microstructures, and mechanical properties (such as strength, hardness, elongation…) of products made from AA5052 aluminum alloy formed via the ISF process with the assistance of ultrasonic vibration.

## Figures and Tables

**Table 1:** Mechanical properties of AA1050, AA1060 and AA5052 aluminum alloys.

Materials	Yield Strength (MPa)	Strength Coefficient K (MPa)	Hardening Exponent n
AA1050	31.4	145.7	0.05
AA1060	56.0	168.0	0.134
AA5052	186.0	338.6	0.22

**Table 2:** Two-level experimental design.

Parameters	Input Parameter Levels	
	Low Level	Hight Level
Step-down size Δ*z* (mm)	0.5	1.5
Feed rate *f* (mm/min)	200	1200
Thickness *t* (mm)	0.5	1.0

**Table 3:** Face-centered CCD design and the parameter levels.

Parameters	Input Parameter Levels		
	Level 1	Level 2	Level 3
Step-down size Δ*z* (mm)	0.5	1.0	1.5
Feed rate *f* (mm/min)	200	700	1200

**Table 4:** Experimental results of paired T-test for *Fy*.

Thickness	Condition	Mean (N)	StDev (N)	Difference Estimate	95% CI for *μ*_Difference	*p*-Value
*t* = 0.5 mm	UISF	29.18	14.17	−22.54	(−25.52, −19.55)	0.000
	ISF	51.72	19.56			
*t* = 1.0 mm	UISF	111.1	92.6	−90.40	(−126.5, −54.2)	0.000
	ISF	201.4	175.5			

**Table 5:** Experimental results of paired T-test for *Fz*.

Thickness	Condition	Mean (N)	StDev (N)	Difference Estimate	95% CI for *μ*_Difference	*p*-Value
*t* = 0.5 mm	UISF	103.71	47.84	−56.95	(−62.15, −51.75)	0.000
	ISF	160.66	61.42			
*t* = 1.0 mm	UISF	553.60	135.40	−338.80	(−374.10, −303.60)	0.000
	ISF	892.50	190.70			

**Table 6:** Two-level experimental results for *RFz* (%) and *RFy* (%).

StdOrder	RunOrder	Δ*z* (mm)	*f* (mm/min)	*t* (mm)	Average of *RFz* (%)						Average of *RFy* (%)					
					Path 1	Path 2	Path 3	Path 4	Path 5	*RFz*	Path 1	Path 2	Path 3	Path 4	Path 5	*RFy*
5	1	0.5	200	1.0	50.8	50.7	44.0	31.1	33.6	42.0	64.7	66.2	65.2	63.6	61.7	64.5
3	2	0.5	1200	0.5	70.8	70.1	63.0	60.2	61.8	65.2	73.9	69.6	67.4	62.8	64.5	67.3
4	3	1.5	1200	0.5	60.6	48.9	52.2	39.5	30.1	46.2	81.5	74.2	72.0	64.5	64.5	71.9
8	4	1.5	1200	1.0	38.9	40.7	44.4	44.2	49.4	43.5	63.6	53.2	55.6	53.4	44.8	54.0
6	5	1.5	200	1.0	36.5	45.2	48.4	30.4	38.4	39.8	58.9	51.6	48.3	39.7	37.1	45.1
7	6	0.5	1200	1.0	58.2	60.3	62.5	60.1	56.0	59.4	61.3	66.1	71.7	61.4	65.2	69.4
1	7	0.5	200	0.5	59.8	66.3	54.2	35.2	36.7	50.4	65.4	55.2	44.4	58.7	52.2	55.2
2	8	1.5	200	0.5	60.0	48.5	44.2	37.0	29.1	43.7	76.3	60.9	45.2	38.4	37.4	41.4

**Table 7:** Experimental results of average of *RFz* (%) and *RFy* (%) for 5 paths.

StdOrder	RunOrder	PtType	Blocks	Δ*z* (mm)	*f* (mm/min)	Average of *RFz* (%)		Average of *RFy* (%)	
						*t* = 0.5 mm	*t* = 1.0 mm	*t* = 0.5 mm	*t* = 1.0 mm
8	1	−1	1	1.0	1200	46.4	46.6	67.4	60.6
7	2	−1	1	1.0	200	41.4	38.5	50.8	53.3
9	3	0	1	1.0	700	40.8	38.6	50.0	53.7
3	4	1	1	0.5	1200	65.2	59.4	67.3	69.4
1	5	1	1	0.5	200	50.4	42.0	55.2	64.5
11	6	0	1	1.0	700	40.2	38.9	50.0	52.9
5	7	−1	1	0.5	700	53.3	46.0	64.7	63.0
10	8	0	1	1.0	700	40.8	38.9	50.4	53.9
12	9	0	1	1.0	700	40.0	38.6	50.0	53.3
2	10	1	1	1.5	200	43.7	39.8	41.4	45.1
13	11	0	1	1.0	700	40.1	38.5	50.4	54.7
4	12	1	1	1.5	1200	46.2	43.5	71.9	54.0
6	13	−1	1	1.5	700	43.4	38.7	55.0	48.4

**Table 8:** Optimal results of *RFz* (%).

Thickness	Δ*z* (mm)	*f* (mm/min)	*RFz* (%)	Target (%)	Composite Desirability
*t* = 0.5 mm	0.5	1200	64.1695	66.0	0.929538
*t* = 1.0 mm	0.5	1200	58.7272	60.0	0.940716

**Table 9:** Optimal results of *RFy* (%).

Thickness	Δ*z* (mm)	*f* (mm/min)	*RFy* (%)	Target (%)	Composite Desirability
*t* = 0.5 mm	1.5	1200	71.9874	75.0	0.910365
*t* = 1.0 mm	0.5	1200	69.1703	70.0	0.966621

## Data Availability

The original contributions presented in this study are included in the article; further inquiries can be directed to the corresponding author.
